# Distinct Patterns of Tryptophan Maintenance in Tissues during Kynurenine Pathway Activation in Simian Immunodeficiency Virus-Infected Macaques

**DOI:** 10.3389/fimmu.2016.00605

**Published:** 2016-12-19

**Authors:** Julia L. Drewes, Joshua D. Croteau, Erin N. Shirk, Elizabeth L. Engle, M. C. Zink, David R. Graham

**Affiliations:** ^1^Department of Molecular and Comparative Pathobiology, Johns Hopkins University School of Medicine, Baltimore, MD, USA

**Keywords:** SIV, HIV, immunosuppression, tryptophan, kynurenine, IDO1, spleen

## Abstract

Induction of the kynurenine pathway (KP) of tryptophan (TRP) catabolism has been proposed to contribute to T cell dysfunction during human/simian immunodeficiency virus (SIV) infection *via* depletion of local TRP levels and production of immunomodulatory KP metabolites. However, while changes in TRP and KP metabolites have been observed in plasma, their levels in lymphoid tissues and levels of enzymes downstream of indoleamine 2,3-dioxygenase (IDO1) have been relatively unexplored. We used our SIV-infected pigtailed macaque model to analyze longitudinal changes in KP metabolites and enzymes by gas chromatography/mass spectrometry and NanoString nCounter gene expression analysis, respectively, in spleen and blood compared to changes previously established in brain and CSF. We found that TRP levels were remarkably stable in tissue sites despite robust depletion in the circulating plasma and CSF. We also demonstrated that intracellular TRP reserves were maintained in cultured cells even in the presence of depleted extracellular TRP levels. Kynurenine (KYN), 3-hydroxykynurenine, quinolinic acid, and the KP enzymes all displayed highly divergent patterns in the sites examined, though *IDO1* expression always correlated with local KYN/TRP ratios. Finally, we demonstrated by fluorescence-activated cell sorting that myeloid dendritic cells and cells of monocytic lineage were the highest producers of *IDO1* in chronically infected spleens. Overall, our study reveals insights into the tissue-specific regulation of KP enzymes and metabolites and, in particular, highlights the multiple mechanisms by which cells and tissues seek to prevent TRP starvation during inflammation.

## Introduction

Induction of the kynurenine pathway (KP) of tryptophan (TRP) catabolism is thought to play a major role in immune dysfunction in human immunodeficiency virus (HIV)-infected individuals, even those on combination anti-retroviral therapy (cART) (1–8). While the KP is an important source of the cellular cofactor NAD+, TRP is a rare essential amino acid, and small changes in TRP metabolism can have major consequences. For example, reducing extracellular TRP levels suppresses proliferation of actively dividing cells *via* accumulation of uncharged TRP–tRNAs and subsequent activation of the GCN2 stress kinase pathway (9). This mechanism could suppress patients’ T cell responses to HIV or secondary opportunistic infections. In addition to the consequences of TRP depletion, increased TRP catabolism down the KP also leads to generation of metabolites that have immunomodulatory capabilities, which include prevention of T cell proliferation [kynurenine (KYN) and picolonic acid] (10); conversion of naïve T cells or proinflammatory Th17 cells into regulatory T cells following interactions at the aryl hydrocarbon receptor, which may increase susceptibility to opportunistic infections at mucosal surfaces [KYN and 3-hydroxy anthranilic acid (3HANA)] (2, 11); and direct apoptosis of activated T cells (3HANA and cinnabarinic acid) (12, 13).

Thus, both the generation of immunosuppressive KP metabolites and depletion of TRP during HIV/simian immunodeficiency virus (SIV) infection could contribute to peripheral HIV pathogenesis. However, several key questions remain unanswered in the field. For example, while TRP and KP metabolites in the blood of HIV-infected patients have been extensively measured (1, 2, 5–8, 14), whether or not these measurements in the circulation accurately reflect changes occurring in lymphoid tissues is unknown, leading to assumptions that this is a major mechanism of suppression in lymphoid tissues. Additionally, while expression of the rate-limiting enzyme indoleamine 2,3-dioxygenase 1 (IDO1) has been repeatedly examined in the context of HIV/SIV infection, the regulation of downstream enzymes of the KP has been relatively unexplored.

To explore these questions, we measured the longitudinal kinetics of KP enzymes and metabolites by multiplexed mRNA (Nanostring nCounter) and GC-MS/MS assays in spleen and plasma from an accelerated pigtailed macaque model of HIV infection in which animals develop clinical symptoms of AIDS within 3 months (15). Our results show that individual cells (macrophages) and tissues (spleen) actively maintain internal TRP levels while depleting extracellular and circulating (plasma) TRP during KP activation. These data suggest that immunosuppressive effects of IDO1 in tissues are more likely a result of immunomodulatory KP metabolites than TRP starvation, representing a potential paradigm shift in our understanding of IDO1-mediated immunosuppression in HIV pathogenesis.

## Materials and Methods

### Animals

Macaque experiments performed in this study utilized archived samples from juvenile pigtailed macaques (*Macaca nemestrina*) that were either mock inoculated or inoculated intravenously with a neurotropic clone (SIV/17E-Fr) and an immunosuppressive swarm (SIV/DeltaB670), as previously described (16). Animals were euthanized during various stages of disease: acute (7, 10, 14 days p.i.), asymptomatic (21 dpi), and chronic infection (35, 42, 56, and 84 dpi). Animals euthanized prior to their planned euthanasia date due to presentation of clinical symptoms (17) were grouped with 84 dpi animals as SIV terminal (“T” in figures). Blood draws were taken prior to infection as well as on days 7, 10, 14, 21, 28, and every 2 weeks thereafter postinfection. All animals were euthanized in accordance with federal guidelines and institutional policies. Macaques were perfused with sterile PBS while under deep anesthesia, and tissues were collected and flash frozen. All animal studies were approved by the Johns Hopkins Animal Care and Use Committee, and all animals were humanely treated in accordance with federal guidelines and institutional policies.

### Macrophage Cell Culture

Pigtailed macaque peripheral blood mononuclear cells (PBMCs) were cultured in macrophage differentiation media (MDM) containing DMEM supplemented with 20% human AB serum (Gemini Bio Products), 50 ng/mL recombinant human macrophage colony-stimulating factor (R&D Systems), 2 mM sodium pyruvate (Sigma), 2 mM l-glutamine (Life Technologies), 1 mM HEPES buffer (Life Technologies), and 20 µg/mL gentamicin (Gibco) for 7 days until mature macrophages were visible. Macrophages were then washed 3× with PBS and switched to MDM with 10% human AB serum. Twenty-five nanograms per milliliter of rhesus IFNγ (R&D Systems) or 400 U/mL human IFNβ1a (PBL Interferon Source) were added in duplicate wells for each time point. After 0, 24, 48, and 72 h, supernatants were saved for KP metabolite analysis, and cells were washed and then harvested with gentle scraping for KP metabolite and *IDO1* gene expression analysis. Metabolites were normalized to controls at each time point as well as to tyrosine (TYR), which does not change during HIV infection (18), to control for minor variations in cell number that could affect metabolism.

### Metabolite Extraction from Spleen

A 3-mm punch was taken from perfused, snap-frozen spleen tissue from SIV-infected pigtailed macaques. Punches were weighed and diluted 1/20 (weight/volume) with cold 0.1% ascorbic acid and sonicated with a tip sonicator three to five times at 70% amplitude for 5 s each, in batches on ice. Residual debris was removed by brief tabletop centrifugation prior to extraction of metabolites. On ice, 50 µL of spleen homogenates (of the original 1/20 homogenates for metabolite measurement, or diluted further for a final of 1/2,000 in 0.1% ascorbic acid for amino acid measurement) were spiked with 50 µL of a solution containing the following heavy standards: [^2^H_5_] TRP (1 µM, CDN Isotopes, QC, Canada), [^2^H_2_] TYR (1 µM, CDN Isotopes, QC, Canada), [^2^H_6_] KYN (5 µM, custom synthesis from Sigma-Aldrich Isotec), and [^2^H_3_] quinolinic acid (0.05 µM, custom synthesis from Synfine, ON, Canada). Fifty microliters of ice cold acetone were added to homogenates and samples were centrifuged at 20,000 *g* for 5 min to precipitate protein. Eighty percent (120/150 μL) of the acetone supernatant was transferred to a new microcentrifuge tube, to which 50 µL of a 2:5 ratio methanol:chloroform was added. Samples were centrifuged at 20,000 *g* for 10 min, and 60% (80/134 μL) of the aqueous layers were transferred to glass vials. The aqueous solution was dried and then derivatized with 120 µL of 2,2,3,3-pentafluoro-1-propanol (Sigma-Aldrich) and 135 µL of pentafluoropropionic anhydride (Sigma-Aldrich) by heating at 75°C for 30 min. Derivatized samples were dried and stored at −80°C.

### Metabolite Extractions from Plasma and Macrophage Cell Culture Supernatants

Metabolite extractions from plasma and cell culture supernatants were identical to spleen with the following modifications: for plasma samples, 2 µL of cell-free plasma were diluted 1/25 with 0.1% ascorbic acid in a final volume of 50 µL for metabolite and amino acid measurements. For cell culture supernatants, cell-free supernatants were diluted 1/2 in 0.2% ascorbic acid for metabolites and 1/100 in 0.1% ascorbic acid for amino acids, all in a final volume of 50 µL. The heavy standard mixture for plasma and cell culture supernatant matrices required higher concentrations for two metabolites: 15 µM instead of 5 µM for [^2^H_6_] KYN, and 0.2 µM instead of 0.05 µM for [^2^H_3_] quinolinic acid. All other heavy standards were kept at 1 µM. Acetone extraction supernatants were dried down directly on a speedvac without methanol/chloroform extraction and then derivatized as above.

### Metabolite Extraction from *In Vitro* Macrophage Cells

Macrophage cell pellets were resuspended in 100 µL of 0.1% ascorbic acid and sonicated 2× for 3 s each at 30% amplitude with a tip sonicator on ice. Fifty microliters of this homogenate were used as the starting material for intracellular amino acid and metabolite measurements. The heavy standard concentrations were identical to those used for plasma and cell culture supernatant samples. The acetone precipitation, methanol/chloroform extraction, and derivatization for the macrophage cell pellets were otherwise performed in an identical manner to the protocol used for spleen homogenates.

### GC/MS/MS Analysis of Amino Acids and KP Metabolites

Dried samples were resuspended in 50 µL ethyl acetate, with the exception of the *in vitro* macrophage lysates, which were resuspended in a smaller volume of 25 µL to maximize signal. One microliter, or 2 µL for the *in vitro* macrophage lysates, was injected in pulsed, splitless mode into an Agilent 7890A GC coupled to a 7000 MS/MS equipped with an Agilent 7693A autosampler operated in electron capture negative ionization mode, as previously described (19). The multiple reaction monitoring transitions used in this study were based on transitions initially described by Eckstein et al. (20) and have been subsequently optimized. The transitions for the derivatized compounds were as follows: TRP Q1 = 608.2, Q3 = 270, CE = 5, retention time (RT) = 13.1 min; [^2^H_5_] TRP Q1 = 613.2, Q3 = 351, CE = 20, RT = 13.1 min; TYR Q1 = 585.2, Q3 = 268, CE = 20, RT = 10.9 min; [^2^H_2_] TYR Q1 = 587.2, Q3 = 436.9, CE = 20, RT = 10.9; KYN Q1 = 612, Q3 = 442, CE = 10, RT = 13.0 min; [^2^H_6_] KYN Q1 = 618, Q3 = 447, CE = 10, RT = 13.0 min; QUIN Q1 = 431.0, Q3 = 298.0, collision energy (CE) = 10 V, RT = 9.4 min; [^2^H_3_] QUIN Q1 = 434.0, Q3 = 301.0, CE = 10 V, RT = 9.4 min; 3HK Q1 = 630, Q3 = 219, CE = 15, RT = 12.9 min. Data were analyzed in a blinded fashion using the Agilent MassHunter software, Build B.04 or B.05. Samples were injected in a randomized order to minimize experimental bias. Each sample was injected at least twice. The average of the peak areas from replicate injections were normalized to the average of the heavy standards for each respective compound. 3HK was the only compound for which we did not have a matching heavy standard; thus, we normalized 3HK to [^2^H_6_] KYN because of their similar ionization properties. These values were fit to the matrix-spiked light standard curves for each compound based on the standard addition method.

### Flow Cytometry

Single-cell suspensions of spleen were obtained by mechanical disruption and passage through a 100 µM filter. Cells were then stained for fluorescence activated cell sorting (FACS) of dendritic, monocytic, and T cell populations, examined on a FACSAriaII (BD) at the SKCCC Flow Cytometry Core, or were stained with separate panels for extensive flow cytometry phenotyping on an LSRFortessa (BD). Representative gating is shown in Figures S1 and S2 in Supplementary Material, respectively, and a list of macaque antibody clones is provided in Table S1 in Supplementary Material. Data were analyzed using the FACSDiVa 6.1.3 (BD) or FlowJo (FlowJo) software.

### qRT-PCR

RNA was extracted using the RNeasy Plus Mini kit (Qiagen). RNA was reverse transcribed using the High Capacity cDNA Kit (Invitrogen Life Technologies; FACS-sorted spleen cells) or SuperScript III (Invitrogen Life Technologies; PBMCs and *in vitro* macrophages) on a thermal cycler: 25°C for 10 min, 37°C for 120°C min, and 85°C for 5 min.

Because of the small amount of starting material in the sorted cells, cDNA from FACS samples were pre-amplified using the TaqMan PreAmp Master Mix (Life Technologies) with a pooled assay of 0.2× primers for *GAPDH* and *IDO1* on a thermal cycler: 95°C for 10 min, 95°C for 15 s, 60°C for 4 min, with steps 2–3 repeated 14 times. Preamplified cDNA was then diluted 1/20 and amplified again using the TaqMan Gene Expression Assay (Life Technologies).

All other cDNA samples were amplified using the Multiplex NoRox PCR Mix (Qiagen): 50°C for 2 min, 95°C for 10 min, 95°C for 15 s, 60°C for 1 min, with steps 3–4 repeated 40 times. *GAPDH* VIC primer/probe was obtained from Life Technologies (Rh02621745_g1). *18S* and *IDO1* primer/probes were custom synthesized from Integrated DNA Technologies: *IDO1* (5′-TGCTTTGACGTCCTGCTGG, 5′-TTCCTGTGAGCTGGTGGCA, and 5′-TXR—ATGCTGCTCAGTTCCTCCAGGACA —IAbRQs); *18S* rRNA (5′-TAGAGGGACAAGTGGCGTTC, 5′-CGCTGAGCCAGTCAGTGT, and 5′-Cy5—AGCAATAACAGGTCTGTGATG—BHQ2). Results were analyzed *via* the 2^−ΔΔCt^ method, with similar results following normalization to either *18S* or *GAPDH*.

### NanoString Gene Expression Analysis

As part of a larger collaborative project, 200 ng of RNA from spleens from 58 animals euthanized at various time points postinfection were analyzed *via* a custom NanoString CodeSet for 116 macaque genes based on rhesus macaque (*Macaca mulatta*) and human sequences, as previously described (21). RNA was harvested from 3-mm punches of snap-frozen macaque spleen tissue by homogenization in RNA STAT-60 (Isotex Diagnostics) in lysing matrix tubes followed by RQ1 DNase treatment (Promega) for 1 h at 37°C and purification with the miRVana Kit (Invitrogen). Target sequences used in this study are listed in Table S2 in Supplementary Material. RNA was hybridized for 16 h using the CodeSet and analyzed using the nCounter Digital Analyzer. Data were normalized to the geometric mean of six spiked-in positive controls to correct for assay efficiency. The background signal threshold was defined as the average of eight spiked-in negative controls +2 SDs following positive control correction, a threshold of 30 counts. None of the genes in this study had a majority of counts below this threshold. As no single housekeeping gene has been proven to be consistently expressed across different cell types, cell maturation states, tissue types, or disease models (22–26), we performed Kruskal–Wallis non-parametric analyses of variance on all positive control normalized genes to find the most stably expressed genes across the spleen samples (22, 27). We found that the markers *CD19, GEM, STK25*, and *CCS* were the least varying genes between infection groups (Kruskal–Wallis *p*-values = 0.203, 0.170, 0.121, and 0.111, respectively). The geometric mean of these four genes was used to normalize the positive control corrected data (26). Trends in SIV viral load and *IDO1* mRNA expression in spleen were independently verified for subsets of the animals by qRT-PCR.

Samples from colon and ileum from 8 additional pigtailed macaques (3 procedural controls and 5 chronically infected animals euthanized at approximately 84 days postinfection) were analyzed in a separate Nanostring CodeSet panel involving 249 macaque genes based on *M. mulatta* and human sequences, as previously described (28). RNA was extracted from 3-mm punches of snap-frozen macaque colon and ileum tissue in a manner identical to the spleen above. Colon and ileal RNA were supplemented with SUPERase-In RNase Inhibitor for long-term storage (1 U/μL final concentration; Life Technologies). Target sequences used for the colon and ileum tissue in this study are listed in Table S3 in Supplementary Material. Colon and ileum transcript RNA data for the five KP genes of interest were normalized to positive control corrected data and then to the geometric mean of four housekeeping genes that were not significantly different between infection groups in the colon or ileum by Kruskal–Wallis: *HPRT1, RPL13A, RPS9*, and *SDHA*.

### Statistical Analyses

Spleen metabolite data were analyzed by Mann–Whitney test to compare peak values during acute infection (the time point with the highest median between days 0 and 14 p.i. for each metabolite, or nadir for TRP), values during asymptomatic infection (day 21 p.i.), and all values during chronic infection (between days 35 p.i. and terminal) against uninfected controls. *p*-values were Bonferroni adjusted for multiple comparisons.

For plasma metabolites, Wilcoxon signed-rank tests were performed on the fold change data to determine whether the peak (nadir for TRP) for each animal during acute infection, or median values for each animal during asymptomatic and chronic infection, were significantly different from their respective preinfection values (set as the theoretical value of 1). *p*-values were Bonferroni adjusted for multiple comparisons.

Macrophage metabolites and *IDO1* enzyme expression data following interferon stimulation were analyzed by Friedman’s test for non-parametric, repeated measures data. Longitudinal spleen Nanostring nCounter mRNA data were analyzed by Kruskal–Wallis test followed by Benjamini–Hochberg false discovery rate corrections to account for the number of genes examined in the assay. Transcripts that had a significant overall Kruskal–Wallis *p*-value <0.05 following Benjamini–Hochberg correction underwent Mann–Whitney tests as described above for spleen metabolites. Colon and ileum Nanostring nCounter data comparing uninfected and terminally infected animals were analyzed by Mann–Whitney test.

Multiple comparison corrections were performed in Microsoft Office 2011 Excel. GraphPad Prism 6.0 was used to perform all other statistical analyses.

## Results

### Longitudinal Kinetics of KP Metabolites in Plasma during SIV Infection

**Figure 1:**
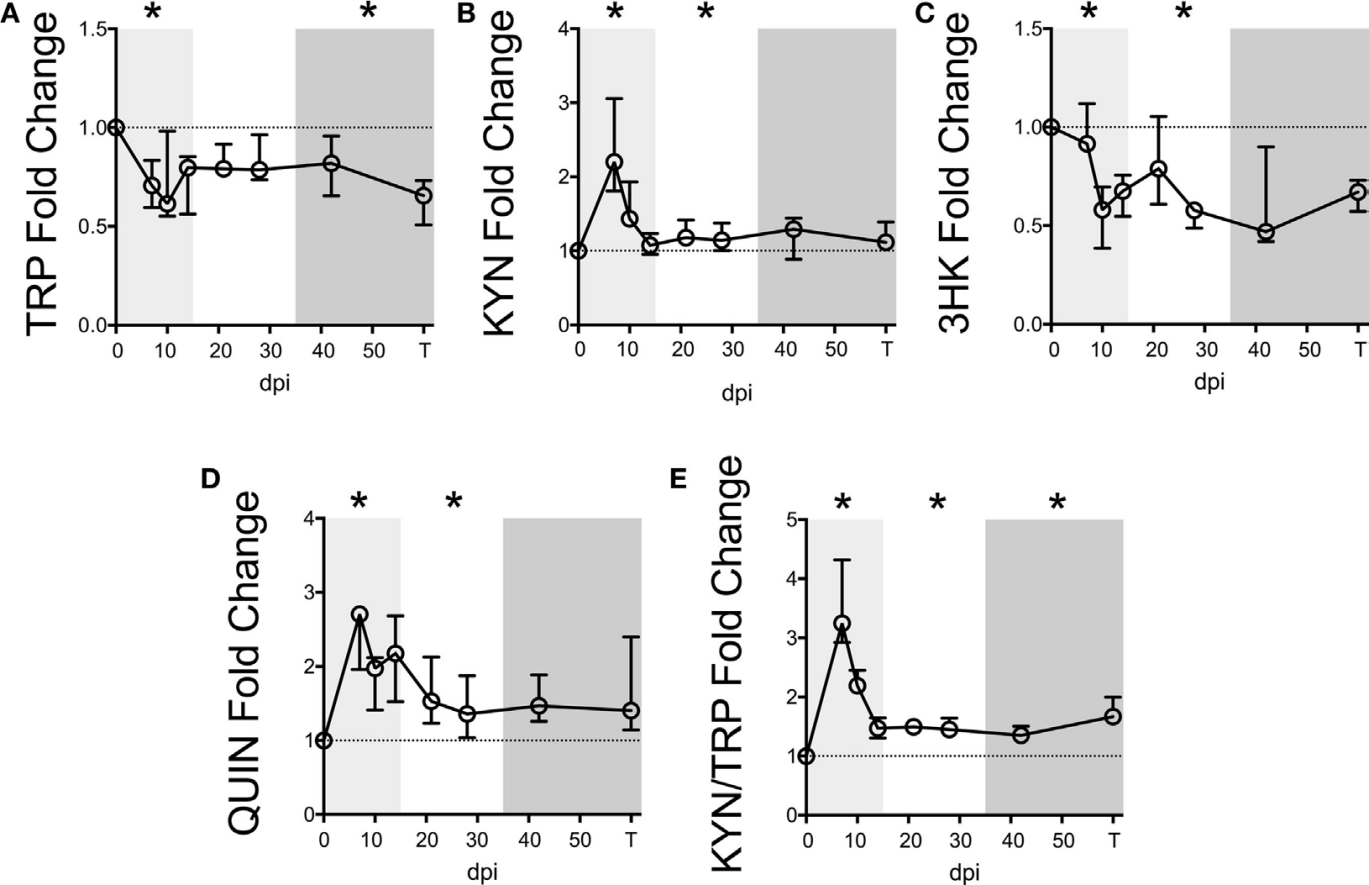
**Longitudinal kynurenine pathway metabolites in plasma during SIV infection of pigtailed macaques**. Seven pigtailed macaques were followed longitudinally, with three plasma samples taken prior to infection and seven samples taken postinfection at days 7, 10, 14, 21, 28, 42, and terminally (approximately 84 dpi). Plasma samples were analyzed for levels of **(A)** TRP, **(B)** KYN, **(C)** 3HK, **(D)** QUIN, and **(E)** KYN/TRP ratios. Data are represented as median fold change over the average of the preinfection samples for each animal. Error bars represent interquartile ranges. Dotted lines represent the median of preinfection values. Light gray shading represents the acute phase of infection (0–14 dpi), and dark gray shading represents the chronic phase of infection (≥35 dpi) in our model. Asterisks (*) indicate whether the peak for each animal during acute infection or median values for each animal during asymptomatic and chronic infection was significantly different (*p* < 0.05) from respective preinfection values by Wilcoxon signed-rank test followed by Bonferroni correction.

We first measured KP metabolites in plasma sampled longitudinally from seven macaques following SIV infection. Baseline levels are listed in Table S4 in Supplementary Material. Plasma TRP displayed an immediate drop during acute infection (Figure 1A, *p* = 0.048 nadir at day 10 p.i. compared to preinfection values), which was sustained throughout asymptomatic and chronic infection, significantly so in the latter (*p* = 0.048).

Levels of KYN, the first major TRP metabolite in the KP, inversely mirrored TRP, peaking significantly at day 7 p.i. (Figure 1B, *p* = 0.048), recovering to baseline by day 14 p.i., and then resurging during asymptomatic infection (*p* = 0.048). In contrast, 3HK, the downstream metabolite of KYN, paralleled TRP and was significantly below preinfection values during acute and asymptomatic infection (Figure 1C, *p* = 0.048 for both analyses). The drop in 3HK levels did not reflect a lack of downstream KP activity, however, as levels of the terminal KP metabolite QUIN were significantly elevated above baseline during acute and asymptomatic infection (Figure 1D, *p* = 0.048 for both comparisons). The KYN/TRP ratio was significantly elevated throughout all three phases of infection compared to preinfection values (Figure 1E, *p* = 0.048 for all phases).

Serum albumin, which binds >90% of total TRP in the blood, was also measured longitudinally in two of the animals. A loss in total TRP concentrations along with stable albumin levels would result in decreased free TRP available for cellular uptake, as cells can only access unbound, free TRP. However, consistent with reports of decreased albumin levels in patients (29–31), we found that serum albumin levels decreased longitudinally alongside total TRP in these two animals, potentially minimizing the impact on free TRP levels (Figure S3 in Supplementary Material).

### Longitudinal Kinetics of KP Metabolites in Spleens of SIV-Infected Macaques

To determine whether TRP and KP metabolites were similarly altered in lymphoid tissue during the course of SIV infection, we harvested metabolites from archived spleens from 58 animals euthanized at various points throughout infection. Exact numbers of animals per time point are listed in Table S5 in Supplementary Material.

**Figure 2:**
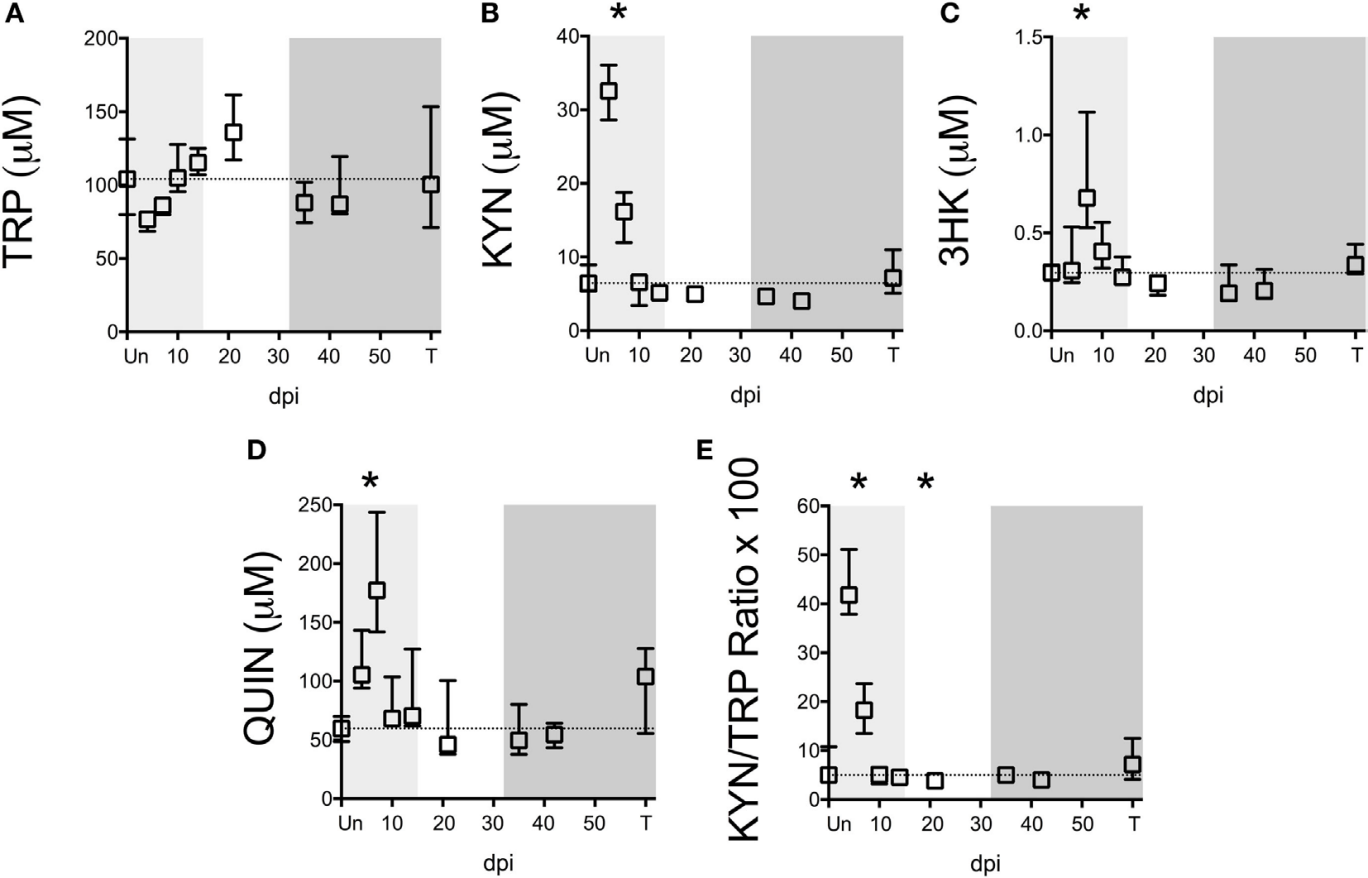
**Longitudinal analysis of kynurenine pathway (KP) metabolites in spleens from SIV-infected pigtailed macaques**. Archived spleen samples from 58 animals euthanized at various points during infection (ranging from 5 to 9 animals per time point) were analyzed for levels of tryptophan (TRP) and KP metabolites: **(A)** TRP, **(B)** kynurenine (KYN), **(C)** 3-hydroxykynurenine (3HK), **(D)** quinolinic acid (QUIN), and **(E)** KYN/TRP ratio × 100. Dotted lines represent the medians of uninfected controls for each metabolite. Light gray shading represents the acute phase of infection (0–14 dpi), and dark gray shading represents the chronic phase of infection (≥35 dpi) in our model. Asterisks (*) indicate whether the peak during acute infection or the total values during asymptomatic or chronic infection was significantly different (*p* < 0.05) from uninfected controls by Mann–Whitney *U* test followed by Bonferroni correction.

Notably, TRP levels did not significantly change at any time point during infection in the spleen (Figure 2A). However, an acute (not significant) drop in median spleen TRP levels at day 4 p.i. from approximately 100 to 70 µM corresponded with an acute increase in KYN levels of approximately 30 µM at this time point that was significant (Figure 2B, *p* = 0.012), indicating a nearly perfect 1:1 turnover of TRP into KYN. KYN returned to baseline by day 10 p.i. and remained at control levels for the remainder of infection. Levels of the downstream metabolites 3HK and QUIN peaked slightly later than KYN (Figures 2C,D, *p* = 0.012 at day 7 p.i. for both 3HK and QUIN), consistent with a temporal progression of degradation down the QUIN axis of the KP during infection. Spleen KYN/TRP ratios were similarly elevated during acute infection (Figure 2E, *p* = 0.012 at the peak at day 4 p.i.), but actually dropped below baseline during asymptomatic infection due to the apparent hyper-recovery of TRP levels at day 21 p.i. (*p* = 0.012 for KYN/TRP ratios at day 21 p.i.). However, neither TRP nor any of the KP metabolites or KYN/TRP ratios underwent any significant changes chronically in the spleen (day 35 p.i. and onward). In comparison to the plasma, the spleen therefore displayed divergent responses. In particular, plasma KYN/TRP ratios, which have been used to predict tissue KP activation, were found to overestimate tissue KYN/TRP induction.

### Systems-Wide Comparison of KP Changes during SIV Infection

**Figure 3:**
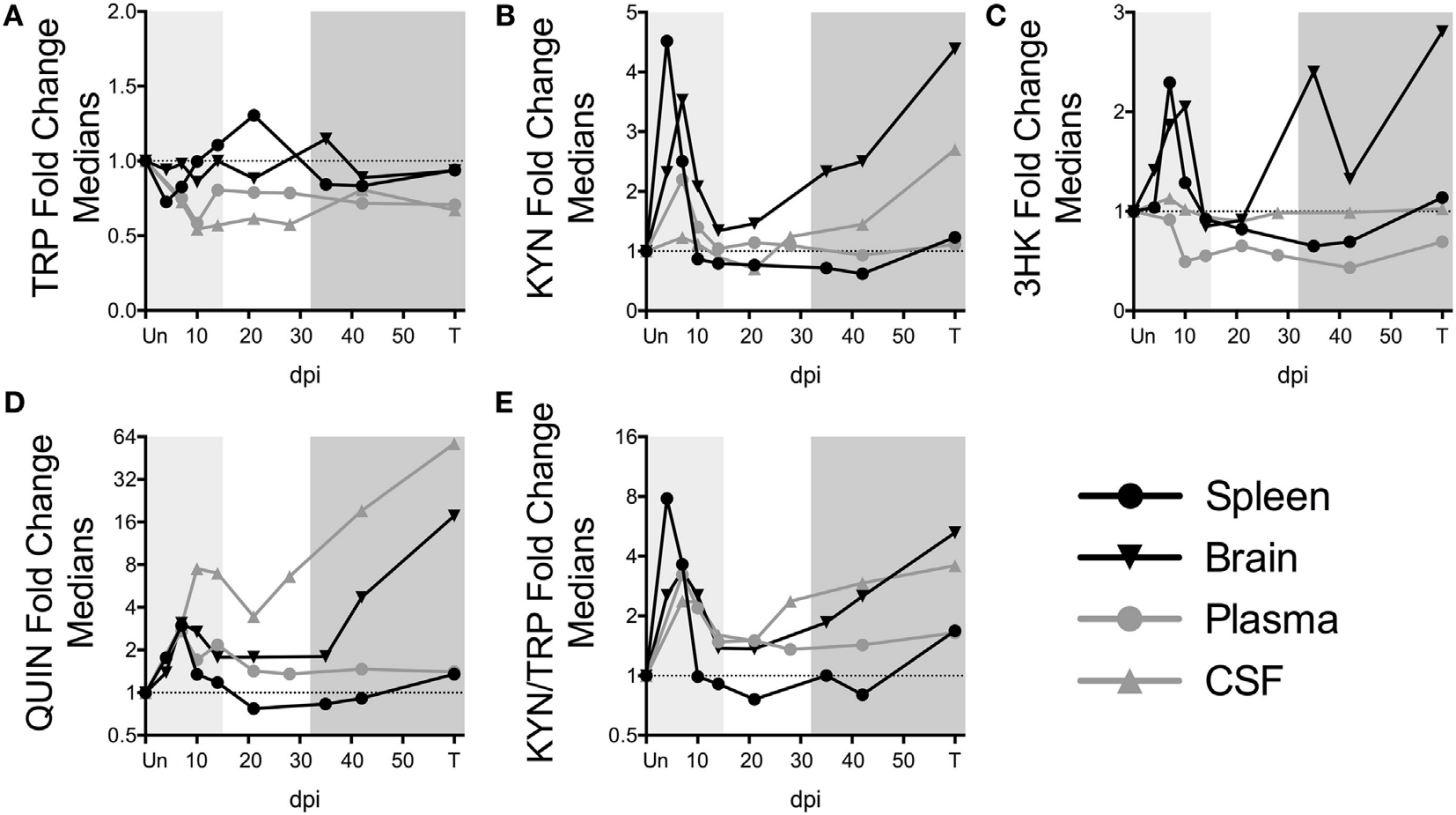
**Comparison of fold changes in kynurenine pathway metabolites in spleen, brain (striatum), plasma, and CSF from SIV-infected pigtailed macaques**. The median fold changes for tryptophan (TRP) **(A)**, kynurenine (KYN) **(B)**, 3HK **(C)**, QUIN **(D)**, and KYN/TRP ratios **(E)** are overlaid for each respective tissue. The horizontal dotted line represents the median of uninfected animals. Light gray shading represents the acute phase of infection (0–14 dpi), and dark gray shading represents the chronic phase of infection (≥35 dpi) in our model. Striatal brain and CSF metabolite data have been modified from a previously published version (32).

To further understand the dynamics of KP flux occurring at different sites in the body, we compared KP metabolites in plasma and spleen with our previous data from CSF and striatal brain tissue from these animals (32) (Figures 3A–E). The striatal region (a component of the basal ganglia) was chosen because it harbors abundant viral replication and demonstrates hallmarks of HIV-associated neurological disease in both HIV and SIV infection, likely in part due to the striatum’s susceptibility to the neurotoxic KP metabolites 3HK and QUIN, as well as other sources of oxidative stress and neurotoxicity (33–36). Comparing the brain vs. the spleen could thus provide a glimpse into commonalities or differences of the diverse tissue morbidities that occur during HIV/SIV infection. TRP was largely stable in the tissues (brain and spleen), even rising above uninfected levels at certain time points despite substantial reductions in the circulating fluids (CSF and plasma) (Figure 3A). In contrast, KYN, 3HK, QUIN, and KYN/TRP levels diverged according to peripheral vs. central lines, with the CNS displaying more robust fold changes than the periphery, particularly during chronic infection (Figures 3B–E). A noticeable second spike in each animal’s plasma QUIN levels at day 14 of acute infection was likely a result of drainage of QUIN from the CSF to the plasma (Figure 3D), given that CSF was the only site that still had highly elevated levels at this time point, highlighting the diversity of tissue sites influencing plasma levels.

### Preservation of Intracellular TRP Levels in Macrophages

**Figure 4:**
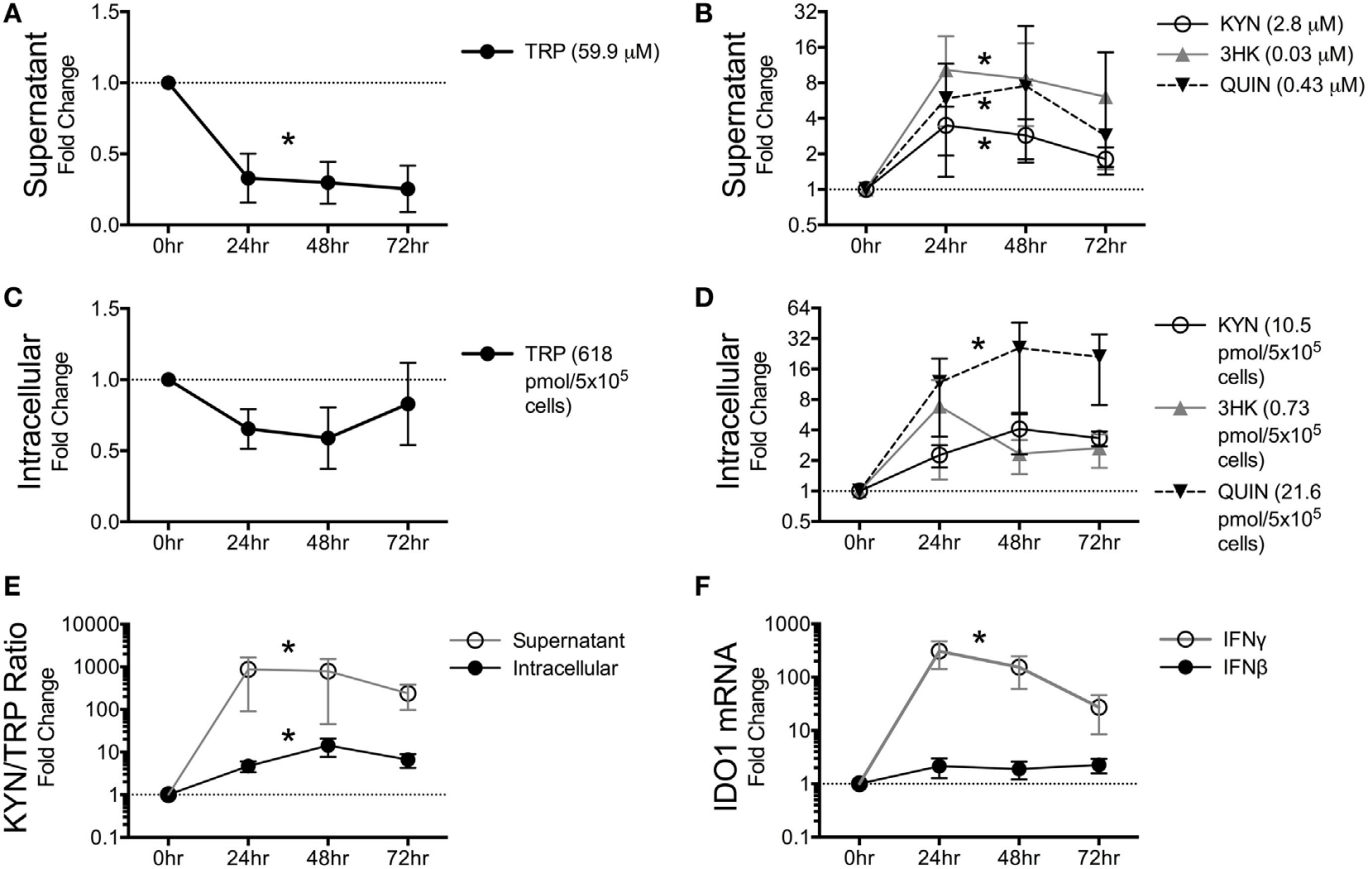
**Macaque macrophages maintain intracellular tryptophan (TRP) levels at the expense of extracellular TRP during kynurenine pathway activation**. Pigtailed macaque macrophages were stimulated with 25 ng/mL IFNγ. After 0, 24, 48, and 72 h, cell-free supernatants were harvested, and cell cultures were halved into aliquots for measurements of mRNA (*IDO1*) and metabolites (TRP, KYN, 3HK, and QUIN). To control for minor well-to-well variations in cell number that could affect metabolic turnover, intracellular and extracellular metabolites were normalized to intracellular and extracellular levels of the amino acid tyrosine, respectively. Data are represented as means ± SEM of the fold change over unstimulated controls at each time point. The average raw values of each metabolite in the unstimulated controls prior to fold change transformation are shown in parentheses on each graph (micromolar for supernatants; pmol/5 × 10^5^ cells for intracellular lysates). The dotted lines represent the unstimulated controls at each time point. Results are from six independent experiments from different macaques, each with two cell culture technical replicates. **(A)** TRP levels in macrophage supernatants following IFNγ stimulation. **(B)** KYN, 3HK, and QUIN levels in macrophage supernatants following IFNγ stimulation. **(C)** Intracellular TRP from harvested cells following IFNγ stimulation. **(D)** Intracellular metabolites KYN, 3HK, and QUIN from harvested cells following IFNγ stimulation. **(E)** Intracellular and extracellular (supernatant) levels of KYN/TRP ratios, a surrogate for IDO1 enzyme activity, in macaque macrophages following IFNγ stimulation. **(F)** Intracellular *IDO1* mRNA expression levels normalized to *GAPDH* mRNA in macrophage lysates following IFNγ or IFNβ stimulation as compared to unstimulated cells. Asterisks (*) represent analytes that were statistically different as a whole across all time points compared to controls as analyzed by the Friedman test for non-parametric, repeated measures data.

Given the robust depletion of TRP in the circulating plasma and CSF and its relative stability in the spleen and brain tissues, we hypothesized that cells within tissues might preserve intracellular levels of TRP at the expense of circulating TRP. Macrophages and other cells types such as dendritic cells (DCs) are capable of suppressing T cell proliferation *in vitro via* IDO-mediated TRP catabolism (37, 38). We chose the macrophage as a representative immune cell type to further study *in vitro* due to its critical importance in both the brain and periphery during HIV/SIV infection: in the brain, macrophages, and the closely related microglia are considered to be the single-most important expressers of IDO1 and downstream KP enzymes due to their ability to produce copious amounts of the neurotoxin and KP metabolite quinolinic acid (39–42). Additionally, a dramatic influx of monocytic lineage cells into both the spleen and the brain has been established during both acute and chronic infection; these cells are thought to be a major driver of HIV/SIV-induced tissue pathology in both sites in conjunction with resident macrophages (43–46). Furthermore, macrophages are thought to be long-lived viral reservoirs in both the spleen and brain, as productively infected macrophages can be isolated from both tissue sites in SIV-infected macaques throughout infection (47). Thus, we stimulated monocyte-derived pigtailed macaque macrophages with IFNγ to model the *in vitro* kinetics of KP induction. Supernatant TRP levels dropped immediately at 24 h and stayed below control levels (Figure 4A, *p* = 0.006). Corresponding to the drop in TRP in the supernatant, the upstream metabolites KYN and 3HK peaked at 24 h, while the terminal pathway metabolite QUIN peaked at 48 h (Figure 4B). All three metabolites increased significantly above controls during the time course (Figure 4B, *p* = 0.037 for KYN, *p* = 0.002 for 3HK, and *p* = 0.009 for QUIN).

In contrast to the immediate and sustained drop in supernatant TRP levels, intracellular TRP suffered only slight losses that did not reach significance (Figure 4C, *p* = 0.256). Some animal donors even displayed slight increases in TRP, while losses in intracellular TRP only occurred in macrophages that had depleted >95% of TRP in the supernatant. The maintenance of intracellular TRP occurred despite modest increases in KYN, 3HK, and QUIN intracellularly (Figure 4D), although only QUIN reached significance (*p* = 0.002). Both supernatant and intracellular KYN/TRP ratios were significantly elevated (Figure 4E, *p* = 0.017 and *p* = 0.006, respectively). In general, fold changes for all of the analytes were greater in the supernatant than in the lysates, suggesting that the cells actively secreted KP metabolites as they were being generated at each step in the pathway and replenished intracellular TRP levels at the expense of the extracellular milieu.

Finally, we determined the kinetics of *IDO1* in response to IFNγ and IFNβ, because chronic stimulation of type I IFN responses by virus is thought to contribute to chronic induction of *IDO1* in HIV/SIV infection in addition to IFNγ (48). However, *in vitro*, IFNγ, but not IFNβ, significantly induced *IDO1* (Figure 4F, *p* < 0.0001 for IFNγ, *p* = 0.772 for IFNβ).

### Kinetics of KP Enzymes during SIV Infection in Spleen

**Figure 5:**
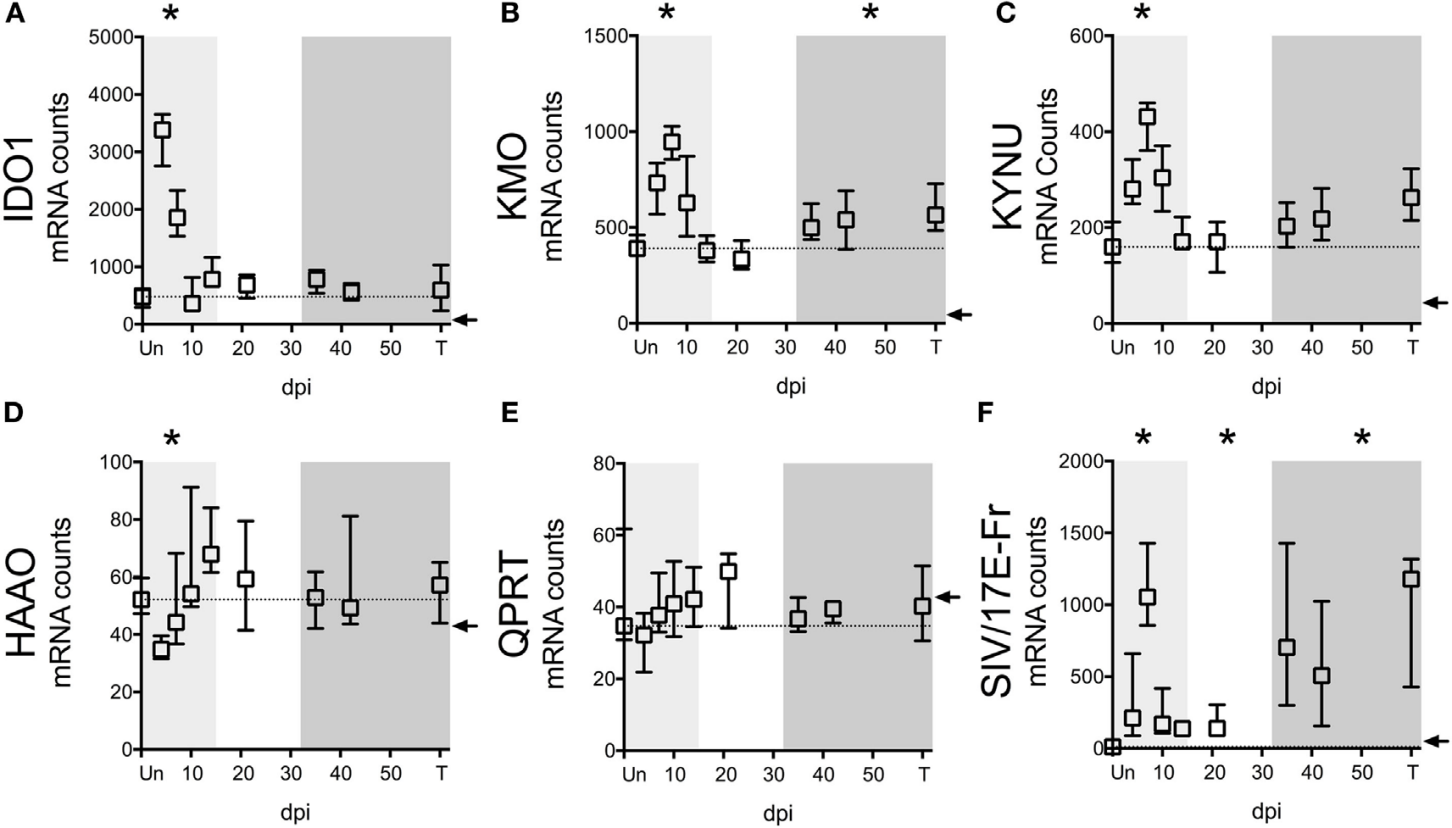
***IDO1* transcriptional regulation mirrors kynurenine (KYN)/tryptophan (TRP) induction in spleens of SIV-infected pigtailed macaques**. mRNA was harvested from spleens of 58 animals euthanized at various points following SIV infection and analyzed for transcriptional expression of SIV RNA and enzymes in the QUIN/NAD+ producing axis of the IDO1 pathway of TRP metabolism. **(A)** Expression of *IDO1*, which mediates the oxidative cleavage of the pyrrole ring of TRP to form l-formylkynurenine, the precursor to KYN. **(B)** Expression of kynurenine 3-monooxygenase (*KMO*), also known as kynurenine 3-hydroxylase, which adds a hydroxyl group to KYN to generate 3HK. **(C)** Expression of kynureninase (*KYNU*), which catalyzes the hydrolytic cleavage of 3HK to form 3HANA and also catalyzes the cleavage of KYN to form anthranilic acid. **(D)** Expression of 3-hydroxyanthranilate-3,4-dioxygenase (*HAAO*; also known as *3HAO*), which oxidatively cleaves the ring of 3HANA to form 2-amino-3-carboxymuconic semialdehyde, the precursor to QUIN. **(E)** Expression of quinolinate phosphoribosyltransferase (*QPRT*), which mediates the conversion of quinolinic acid into NAD+. **(F)** Expression of SIV/17E-Fr *Gag* RNA. The dotted lines in graphs **(A–E)** represent the medians of uninfected controls. Arrows represent the background threshold of the assay. Asterisks (*) represent time points that are significantly different (*p* < 0.05) from uninfected values by Kruskal–Wallis with Bonferroni correction.

In addition to the ability of cells to preserve intracellular levels of TRP, a deficiency in *IDO1* expression could also explain the lack of TRP depletion in tissues. We thus analyzed the expression of *IDO1* mRNA as well as other key enzymes in the KP in spleens of the SIV-infected macaques by NanoString. Paralleling trends in the CNS of these animals (32), we found that upstream enzymes (*IDO1, KMO*, and *KYNU*) displayed significant mRNA upregulation during at least one phase of infection (Figures 5A–C), while downstream enzymes (*HAAO* and *QPRT*) were either downregulated or unchanged (Figures 5D,E). Importantly, *IDO1* was significantly elevated only during acute infection (Figure 5A, *p* = 0.012 at day 4 p.i.), suggesting that while the lack of TRP depletion in the spleen during acute infection was due to tissue preservation of TRP, chronically it was due to a lack of local *IDO1* induction.

**Figure 6:**
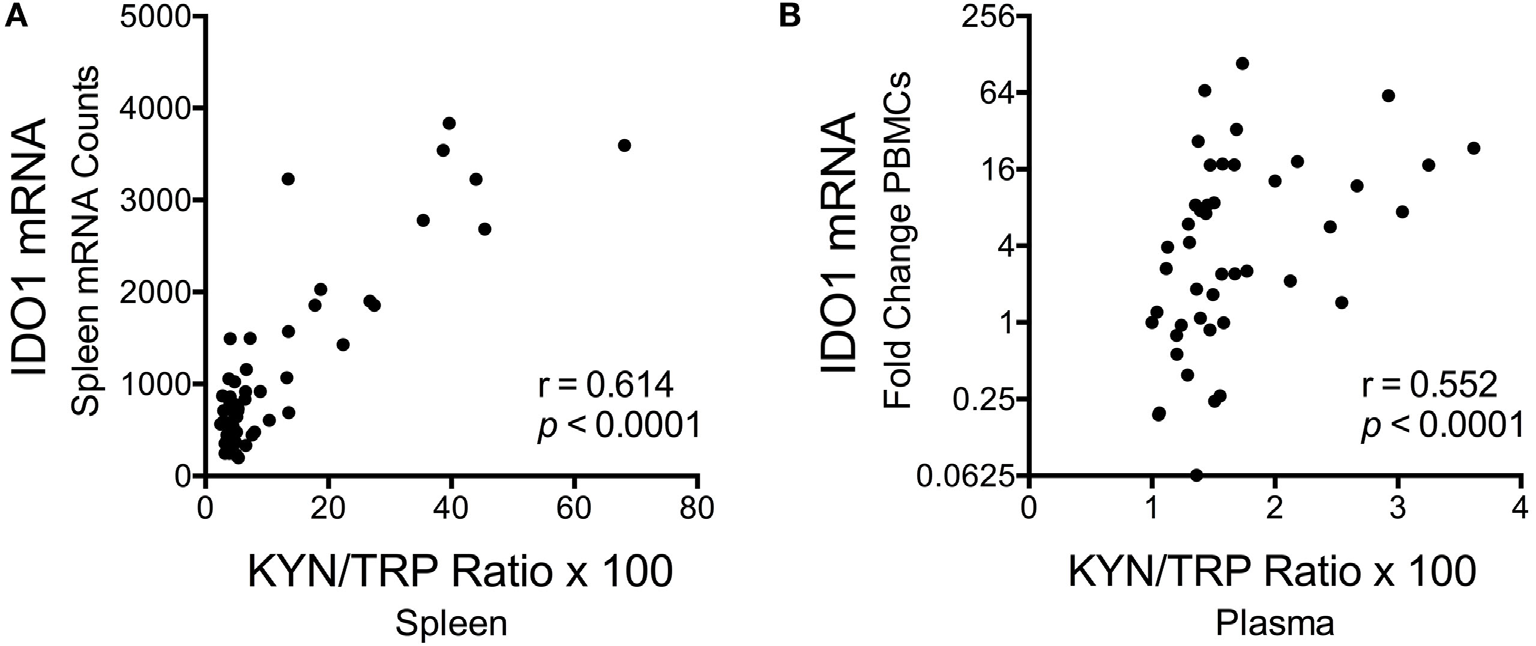
**Correlations between longitudinal kynurenine (KYN)/tryptophan (TRP) ratios and *IDO1* mRNA expression in spleen and blood of SIV-infected pigtailed macaques**. **(A)** Spearman’s rank correlation of splenic *IDO1* mRNA expression and splenic KYN/TRP ratios harvested from animals euthanized at various points postinfection (*n* ≥ 5 animals per timepoint). **(B)** Spearman’s rank correlation of peripheral blood mononuclear cell *IDO1* mRNA expression and plasma KYN/TRP ratios from five animals followed longitudinally (3 preinfection blood draws averaged together, as well as 7 postinfection blood draws at days 7, 10, 14, 21, 28, 42, and approximately 84 dpi, are depicted).

Despite the fact that SIV/HIV can induce *IDO1* both directly and indirectly (49, 50), spleen SIV RNA peaked later than *IDO1* (days 7 vs. 4 p.i.) during acute infection (*p* = 0.012 for SIV at day 7 p.i.), and remained elevated throughout asymptomatic (*p* = 0.012) and chronic infection (*p* < 0.001), suggesting that the lack of *IDO1* induction chronically was not due to a lack of virus (Figure 5F). Finally, spleen *IDO1* expression correlated strongly with spleen IDO1 activity (KYN/TRP ratios) (Figure 6A, *r* = 0.614, *p* < 0.0001). Likewise, *IDO1* mRNA in PBMCs positively correlated with KYN/TRP ratios in the plasma (Figure 6B, *p* < 0.0001, *r* = 0.552). These data confirmed the close relationship between local *IDO1* mRNA levels and local IDO1 enzyme activity.

Notably, the lack of chronic *IDO1* induction in the spleen was not due to a total of peripheral KP activation chronically in our model, as ileal and colonic gut tissue from subsequent animals demonstrated significant upregulation of *IDO1* enzyme mRNA during chronic infection (Figure S4 in Supplementary Material; *p* = 0.024 for ileum, *p* = 0.048 for colon *IDO1* expression terminally compared to uninfected). *KMO* and *KYNU* expression trended upwards during chronic infection in these gut tissues, while *HAAO* and *QPRT* trended downwards, similar to what was observed in the spleen tissue chronically, although none of these trends were statistically significant. The chronic induction of IDO1 that we observed in these gut tissues is consistent with induction observed by others in gut tissue of SIV-infected rhesus macaques and confirms the involvement of the gut in the pervasive immunosuppressive features of chronic SIV infection (3).

### Cell-Specific IDO1 Expression in Chronically Infected Spleen

**Figure 7:**
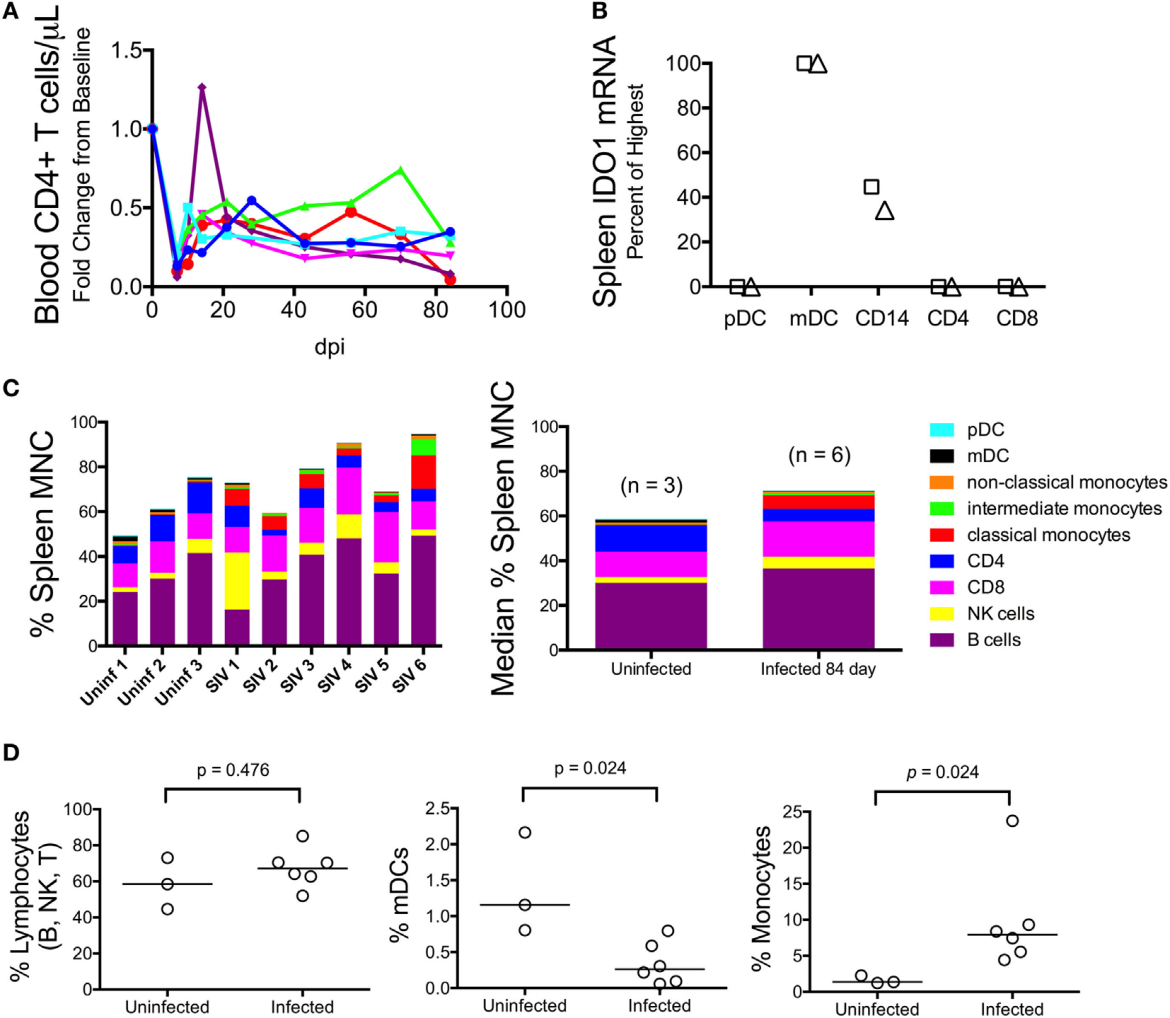
**Myeloid dendritic cells (mDCs) express the highest levels of *IDO1* mRNA in chronically SIV-infected macaque spleen**. **(A)** Absolute CD4+ T cell counts in the blood were measured longitudinally using complete blood count and flow cytometry in five animals to confirm immune suppression. **(B)** Cells were teased out of spleen tissue from two animals (designated as the triangle and square, respectively) at 84 days p.i., then sorted by fluorescence-activated cell sorting to separate pure populations of pDCs, mDCs, CD14+ monocyte/macrophages, CD4+ T cells, and CD8+ T cells. mRNA was extracted from the isolated cells and analyzed for *IDO1* expression levels. All data were normalized to internal *GAPDH* housekeeping mRNA and then to the highest expressing cell type, which was the mDC population in both samples, by the 2^−ΔΔCt^ method. Fold change data were transformed to a percentage of the mDC values. Similar results were obtained with normalization to *18S* ribosomal RNA. **(C)** Mononuclear cells from all five chronically infected animals as well as three uninfected controls were analyzed by an extensive flow cytometry panel for various cell markers. Raw cell percentages are depicted in the left-most panel; median cell percentages are shown in the panel on the right. **(D)** Specific populations of cells were further analyzed by Mann–Whitney test to determine statistical significance.

We next examined whether the lack of *IDO1* induction chronically in the spleen was due to dysfunction or possibly even depletion of antigen-presenting cells (APCs), which are typically the highest *IDO1* expressers, although the cell-specific contributions during HIV/SIV infection are not well established. Splenic mononuclear cells (MNCs) were harvested immediately following euthanasia in a subsequent group of animals. These chronically infected animals were clearly immunocompromised, consistent with our model, as levels of CD4+ T cells in their peripheral blood were well below baseline during chronic infection (Figure 7A). Splenic MNCs from two of these infected animals underwent cell sorting by FACS (see Figure S1 in Supplementary Material for sample gating) and qRT-PCR for cell-specific *IDO1* expression. From these FACS-sorted cells, we found that myeloid dendritic cells (mDCs) and CD14+ monocytes from the chronically infected spleens maintained their ability to express high levels of *IDO1* mRNA (Figure 7B). Our finding that mDCs expressed the highest levels of *IDO1* is in agreement with a previous study in HIV-infected patients in which staining for IDO1 protein in lymph nodes mostly colocalized with an mDC-specific marker (2).

Given that the spleen APCs were capable of expressing *IDO1*, we next compared the proportions of various cell types to one another by flow cytometry to see whether a relative decline in APC number could explain the surprising lack of *IDO1* expression in the chronically infected spleens (representative gating is shown in Figure S2 in Supplementary Material). The analysis revealed that B, NK, and CD8+ T lymphocytes appeared to be present at higher proportions in the spleens of infected animals compared to uninfected animals (Figure 7C). However, due to losses in splenic CD4+ T cells during infection, the total lymphocyte percentage remained the same (Figure 7D, first panel). As for APCs, we observed depletion of mDCs (Figure 7D, second panel; *p* = 0.024) but an increase in monocytes (Figure 7D, third panel; *p* = 0.024). Thus, the *IDO1* deficiency in the spleen was not due to a complete lack of *IDO1* expression from APCs nor did it appear to be due to a total loss of *IDO1*-expressing APCs within the MNC population, although both factors may still contribute to this phenomenon.

## Discussion

Overall, our comprehensive analysis of the KP in various anatomical sites in SIV-infected macaques reveals tremendous insight into the tissue-specific regulation of KP enzymes and metabolites. Despite our hypothesis that inflammation would trigger ubiquitous changes in the KP, the tissues and circulating fluids displayed highly variable patterns in metabolites and enzymes. Most importantly, we did not find any significant reductions in TRP in either the spleen or brain at any time point during infection, despite TRP levels in the plasma and CSF being reduced by up to 50%, on par with changes reported in plasma and CSF of HIV-infected patients (14, 51, 52). Our *in vivo* findings were further supported by *in vitro* experiments in which macrophages stimulated with IFNγ maintained intracellular TRP levels by depleting extracellular TRP.

These data substantiate a growing body of mechanisms by which cells and tissues may subvert TRP losses during KP activation. For example, IFNγ, which is the major inducer of IDO1, also upregulates tryptophanyl-tRNA synthetase (TTS), an enzyme that complexes intracellular TRP with its corresponding TRP–tRNA (53, 54). Thus, cells exposed to IFNγ may maintain or even accumulate intracellular levels of TRP–tRNA complexes during KP activation, protecting them from anergy that occurs when uncharged tRNAs trigger induction of the GCN2 stress kinase pathway (9, 52, 55). Furthermore, given that extracellular levels of TRP must be below 3 µM *in vitro* in order to activate the GCN2 stress pathway in T cells (9), and the fact that we detected levels of TRP in the tissues well above this concentration throughout infection (100 µM for spleen, 20 µM for brain), it seems unlikely that TRP depletion is a major contributor to T cell dysfunction during HIV/SIV infection. These findings question some of the fundamental assumptions regarding the role of TRP metabolism during immunosuppression and suggest that future studies focus more on the KP metabolites themselves.

However, it is important to distinguish between free TRP and total TRP, as >90% of the circulating TRP is bound to albumin in the blood, and only the free TRP is thought to be accessible to cells. Our TRP measurements in this study represent total TRP (i.e., albumin-bound + free TRP). Thus, a 10% change in total TRP could deplete all available free TRP. However, multiple studies have shown that HIV infection is associated with a decrease in serum albumin as well (29–31). We similarly found evidence for serum albumin depletion in our model, and furthermore, found that it paralleled the trends in plasma TRP depletion. These data suggest that changes in free TRP in the plasma may actually be more stable than previously thought, despite the overall losses in total plasma TRP. However, it is still conceivable that in a tissue microenvironment, such as a germinal center, levels of TRP may reach dangerously low nadirs, or that chronic exposure to slightly lower-than-normal levels of TRP may have long-term consequences on T cell function that has not yet been discerned. Additionally, perfusion of tissues used in this study may have masked any potential changes in TRP levels in extracellular spaces. Future studies that shed light on the differences between extracellular and intracellular TRP and their respective impacts on immune dysfunction are warranted.

In addition to KP metabolites, our results also shed light on the transcriptional regulation of KP enzymes during infection. Concordant with our previous data in brain tissue (32), we found that upstream enzymes in the KP (*IDO1, KMO*, and *KYNU*) displayed more upregulation during acute infection than downstream enzymes (*HAAO* and *QPRT*). Intriguingly, we also found that *IDO1* expression in the spleen actually peaked prior to peak spleen viral loads (days 4 vs. 7 p.i.) during acute infection. This peak in *IDO1* at day 4 p.i. coincided with the robust peak in *IFN*β and multiple IFN-stimulated genes (*MxA, IRF7*) recently published in our model (43). In that study, *IFN*α expression also peaked at day 4 p.i. but was less robustly induced, while *IFN*γ and other known inducers of IDO such as *IL6* and *TNF*α displayed a delayed induction as late as days 7 or 14 p.i. (43), suggesting that the complex milieu of factors that can stimulate *IDO1* expression are not synchronized during infection. Others have shown similar findings during acute infection in plasma of SIV-infected macaques, where IDO1 activity (KYN/TRP ratios) peaked before viral loads during acute infection and coincided with an acute spike in type I IFN levels (56). However, type I IFN is not as proficient as type II IFN at inducing *IDO1 in vitro* (57), which we confirmed in our macrophage experiments. Rather than type I IFN being responsible for the induction of *IDO1* during acute infection, it is possible that this earlier peak in *IDO1* before virus instead reflects rapid *downregulation* of *IDO1* by the same mechanisms involved in the contraction of acute type I IFN responses.

A similar mechanism may be at work during chronic infection, when *IDO1* expression appeared to be suppressed despite high levels of spleen viral loads, induction of other KP enzymes (e.g., *KMO*), and presence of mDCs and CD14+ cells capable of expressing *IDO1*. One such potential negative regulator of IDO1 is the suppressor of cytokine signaling (SOCS) family of proteins. SOCS3 in particular has been shown to inhibit Jak/Stat signaling, which is required for *IDO1* expression, and can also directly target IDO1 protein for proteasomal degradation (58). Notably, SOCS3 has been shown to be elevated in tonsillar tissue of HIV-infected patients (59) and in the CNS (60) and spleens of our SIV-infected macaques (43). In the SIV-infected spleens, *SOCS3* expression was shown to be highly expressed not only during acute infection but also throughout asymptomatic and chronic infection, demonstrating a strong potential reason for the suppression of *IDO1* despite abundant virus stimuli during chronic infection (43).

Alternatively, expression of *IDO1* in at least one DC cell type (CD19+ DCs) has been shown to be dependent on CD4+ T cells in a murine leukemia virus model of retroviral infection (61). Thus, the progressive loss and/or exhaustion of CD4+ T cells in the periphery during chronic SIV infection in our model could contribute to a reduced production of IDO1 by CD4+ T cell-dependent DCs (43). However, it is important to note that *IDO1* was significantly upregulated chronically in the colon and ileum, where CD4+ T cell depletion is also known to occur (62). This discrepancy in *IDO1* induction may be a result of differences in the cell types and/or cytokines contributing to *IDO1* levels in different tissue sites. For example, macrophages and microglia are thought to be the major producers of *IDO1* in the brain (41), whereas mDCs may express the highest level of *IDO1* in the spleen (data shown here) and lymph nodes (2) during HIV/SIV infection. Conversely, IFNα subtypes are known to differ in different tissue sites; such differences could modulate the induction of IDO1 or IDO1 modulators (63). Future studies will be necessary in order to examine the cell-specific requirements of IDO and other KP enzyme induction in other peripheral tissue sites and during acute vs. chronic infection.

Finally, the results of our study have implications for the treatment of not only HIV but also other diseases in which IDO1 and the KP are thought to play a role, such as cancer (64). While our results suggest that circulating TRP levels clearly overestimate the extent of TRP depletion in the tissues, the KYN/TRP ratio in the plasma and the QUIN/TRP ratio in the CSF (32) are consequently highly sensitive biomarkers of inflammation and may help to identify patients in need of immunotherapy prior to irreparable tissue damage. Importantly, cART does not fully restore peripheral KP metabolites to uninfected levels even in patients with undectable viremia (1, 65, 66). IDO1 therefore represents a potentially important therapeutic target that could be used in conjunction with cART in HIV-infected patients or in conjunction with chemotherapy in cancer patients (64). However, mixed results have been observed following attempts to inhibit IDO1 with compounds such as the TRP analog, 1-methyl-d-tryptophan (d-1MT). For example, administration of d-1MT in conjunction with ART during SIV infection of rhesus macaques resulted in only a transient improvement in plasma TRP levels but no change in plasma KYN levels (67). Our data suggest that reducing the KP metabolite levels is of greater importance than restoring TRP, as TRP levels are not at a limiting concentration in the tissues at any time point during infection. Boosting immunotherapy in the rhesus macaque model by combining d-1MT with anti-CTLA-4 antibodies and vaccination resulted in severe side effects including acute pancreatitis (68). Additionally, the beneficial effects of d-1MT may be due to side effects independent of IDO1 (69). These non-specific effects and the relatively high micromolar concentrations of d-1MT required for an effect render d-1MT an unlikely candidate for further applications in patients. As a result, second generation inhibitors of IDO1 continue to be developed (64). However, inhibition of IDO1 is likely complex due to the potential rebound effect of newly uninhibited T cells producing IFNγ—a sign that the IDO1 inhibitor is working—that in turn could paradoxically induce IDO1 (67). Nevertheless, several IDO1 inhibitors including indoximod and epacadostat are now undergoing Phase I/II clinical trials in cancer patients in whom induction of IDO and resulting evasion of anti-tumoral immune responses may play a role (70–72). Advances made toward immunotherapy in HIV-infected patients may also benefit cancer patients and vice versa.

In conclusion, our study suggests that cellular TRP depletion is likely not a major component of IDO1’s immunosuppressive capabilities during HIV/SIV infection. In fact, myriad mechanisms exist to stave off TRP starvation, including (1) downregulation of IDO1 expression by SOCS3; (2) upregulation of TTS to allow cells to store TRP complexed to the TRP–tRNA during IFNγ stimulation; (3) reductions in serum albumin during infection, which alleviates deficits in free TRP; and (4) the relatively high levels of TRP in the tissues, making the likelihood that TRP would be depleted below the necessary <3 µM relatively unlikely. Clearly, these findings will need to be confirmed in other models, but nevertheless they shed light on how cells and tissues prioritize TRP use in the face of KP activation.

## Ethics Statement

All animal studies were approved by the Johns Hopkins Animal Care and Use Committee; all animals were humanely treated in accordance with federal guidelines and institutional policies.

## Author Contributions

JD, JC, ES, EE, MZ, and DG designed the experiments. JD, JC, ES, and EE performed the experiments. JD, JC, and ES analyzed the experiments. JD wrote the manuscript with significant input from JC, ES, EE, MZ, and DG.

## Conflict of Interest Statement

The authors declare that the research was conducted in the absence of any commercial or financial relationships that could be construed as a potential conflict of interest.

## Abbreviations

APC, antigen-presenting cell; ART, anti-retroviral therapy; cART, combination anti-retroviral therapy; d-1MT, 1-methyl-d-tryptophan; DC, dendritic cell; FACS, fluorescence-activated cell sorting; GCMS, gas chromatography mass spectrometry; GCN2, general control non-derepressible 2, also known as EIF2AK4; HAAO, 3-hydroxyanthranilate-3,4-dioxygenase, also known as 3HAO; HIV, human immunodeficiency virus; IDO1, indoleamine 2,3-dioxygenase; IFN, interferon; KMO, kynurenine 3-monooxygenase, also known as kynurenine 3-hydroxylase; KP, kynurenine pathway; KYN, kynurenine; KYN/TRP, kynurenine/tryptophan ratio; KYNU, kynureninase; mDC, myeloid dendritic cell; MNC, mononuclear cell; NAD+, nicotinamide adenine dinucleotide; pDC, plasmacytoid dendritic cell; PBMC, peripheral blood mononuclear cell; QPRT, quinolinate phosphoribosyltransferase; QUIN, quinolinic acid; SIV, simian immunodeficiency virus; SOCS3, suppressor of cytokine signaling 3; TRP, tryptophan; TTS, tryptophanyl tRNA synthetase; TYR, tyrosine; 3HANA, 3-hydroxyanthranilic acid; 3HK, 3-hydroxykynurenine.
