# Supplementary material for: Distinct Patterns of Tryptophan Maintenance in Tissues during Kynurenine Pathway Activation in Simian Immunodeficiency Virus-Infected Macaques
Source: Front Immunol. 2016 Dec 19;7:605. doi: 10.3389/fimmu.2016.00605 (PMC5165277; doi:10.3389/fimmu.2016.00605)
Supplement: Supplementary file 1 [file Data_Sheet_1.PDF]

Supplementary Material

Distinct Patterns of Tryptophan Maintenance in Tissues during Kynurenine Pathway Activation in Simian Immunodeficiency Virus-Infected Macaques

Drewes, J.L.,[1] Croteau, J.,[1] Shirk, E.,[1] Engle, E.L.,[1] Zink, M.C.,[1*] Graham, D.R.[1*]

*** Correspondence:** David Graham: dgraham@jhmi.edu; M. Christine Zink: mczink@jhmi.edu

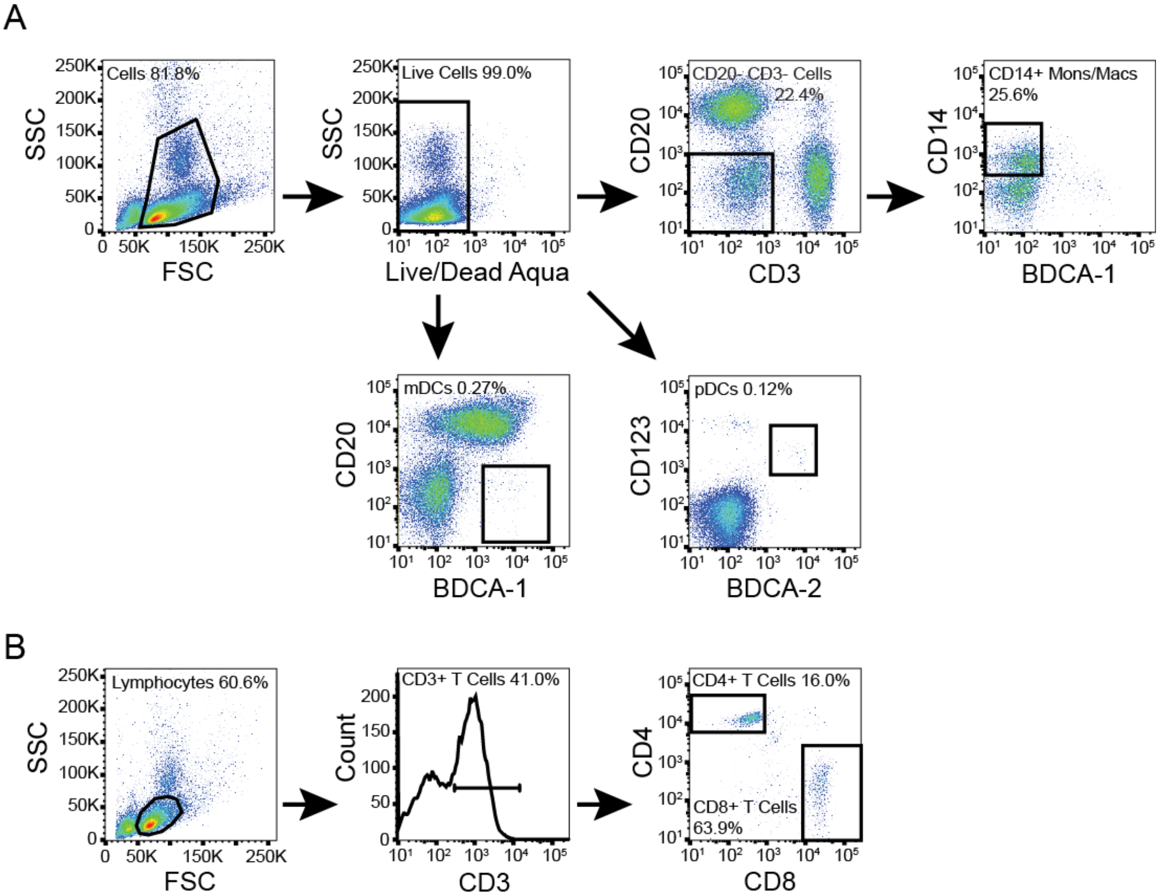

Supplementary Figure 1. Representative FACS gating of splenic mononuclear cells. Splenic mononuclear cells were first gated to include live cells by FSC and SSC gating and live/dead aqua staining. (**A**) CD14+ monocytes (top right) were CD20-/CD3- and CD14+/BDCA1-; mDCs were BDCA1+ and CD20-; pDCs were CD123+/BDCA2+. (**B**) CD4+ lymphocytes were CD3+CD4+, and CD8+ T cells were CD3+CD8+.

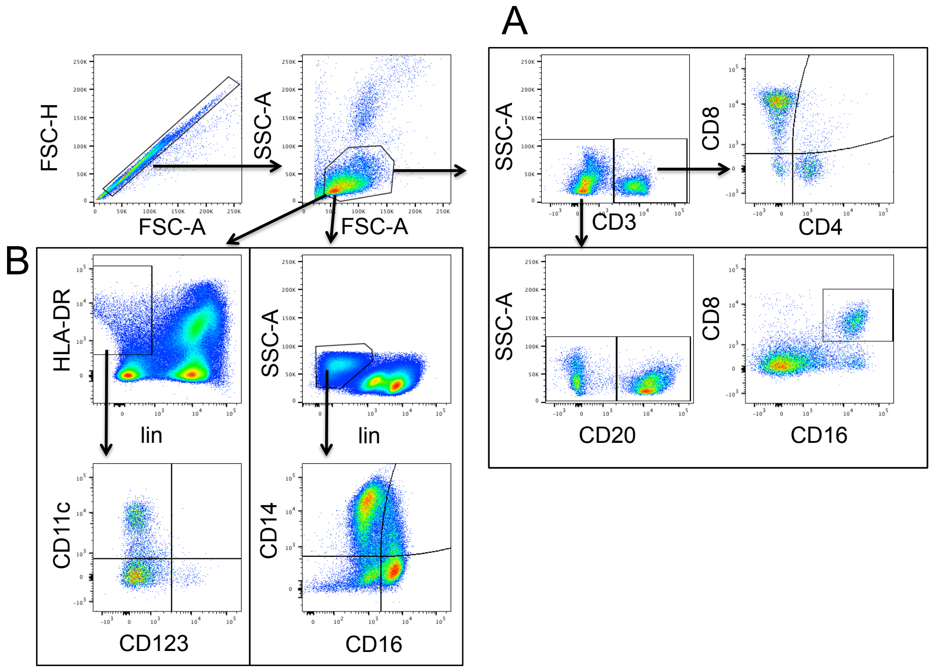

Supplementary Figure 2. **Representative gating for the expanded flow cytometry panel of splenic mononuclear cells**. Cells were first gated to exclude doublets using FSC-A/FSC-H. Mononuclear cells were then gated based on forward and side scatter. (**A**) In the lymphocyte panel, CD3+ cells were further gated as CD4+ or CD8+. CD3- cells were gated as CD20+ B cells or CD8+/CD16+ NK cells. (**B**) In the monocyte/dendritic cell panel, lineage (CD3/CD20/CD8) negative cells were gated and assessed based on their expression of CD14 and CD16. CD14+ CD16- cells were classified as classical monocytes, CD14+ CD16+ cells as intermediate monocytes, and CD14lo/- CD16+ cells as non-classical monocytes. In the dendritic cell panel, mononuclear cells were gated based on negative lineage (CD3/CD20/CD14) markers and positive HLA-DR expression. The HLA-DR+/lin- cells were further gated based on the expression of CD11c for mDCs or CD123 for pDCs.

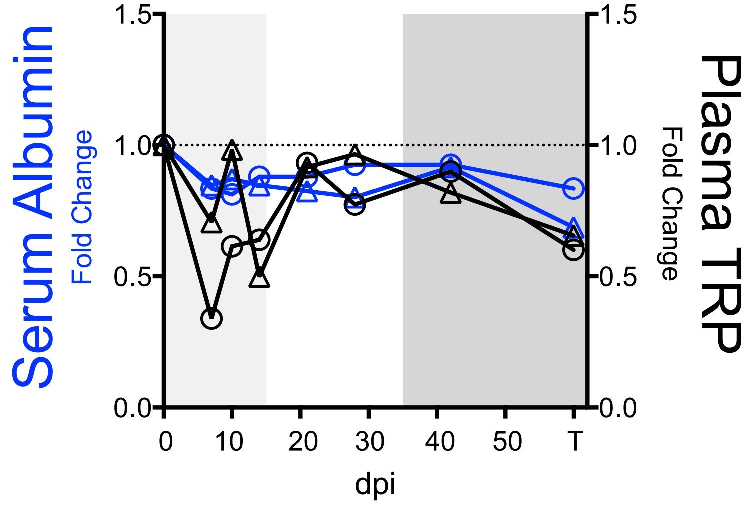

Supplementary Figure 3. Serum albumin levels. Longitudinal serum albumin (blue) and plasma tryptophan (black) fold changes are shown for two SIV-infected macaques, one denoted by circles and the other by triangles.

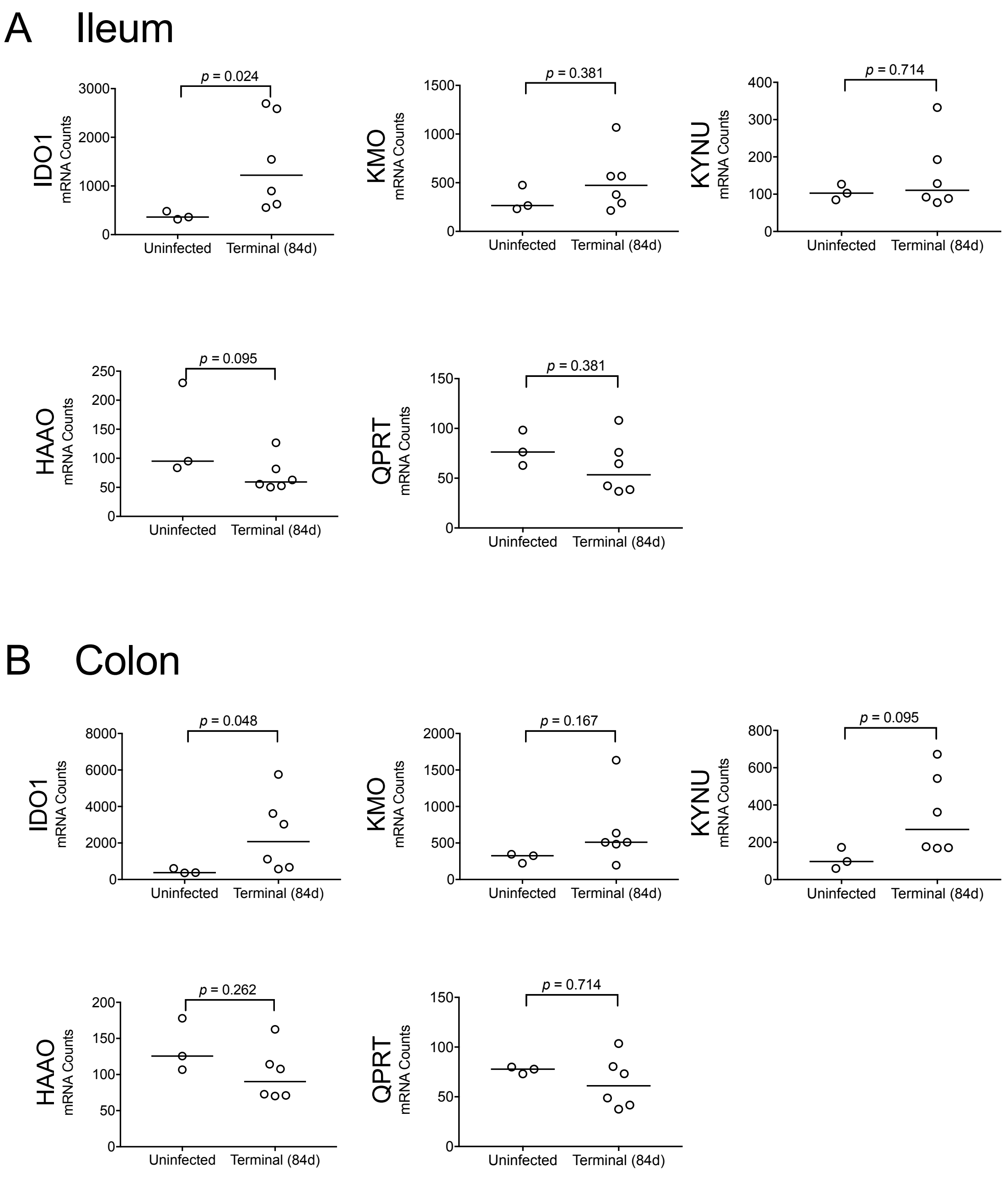

Supplementary Figure 4. Induction of kynurenine pathway enzymes in ileum and colon of chronically infected pigtailed macaques. RNA from (A) ileum and (B) colon of a subsequent group of uninfected (N = 3) and chronically infected animals (N = 5) was extracted and analyzed by Nanostring nCounter analysis (see Methods). Data were analyzed by Mann Whitney.

Supplementary Table 1. Antibodies used for FACS and flow cytometry of macaque spleen.

Panel	Antibody	Fluorophore	Clone	Company
DC Sorting	BDCA-2	APC	AC144	Miltenyi Biotec
DC Sorting	BDCA-1	PE	AD5-8E7	Miltenyi Biotec
DC Sorting	CD123	PerCP-Cy5.5	7G3	BD
DC Sorting	CD20	PE-Cy7	2H7	BD
DC Sorting	CD3	Pacific Blue	SP34-2	BD
DC Sorting	CD14	FITC	M5E2	BD
DC Sorting	Live/Dead	Aqua		Invitrogen
T Cell Sorting	CD3	FITC	SP34-2	BD
T Cell Sorting	CD4	PerCP-Cy5.5	L200	BD
T Cell Sorting	Live/Dead	Aqua		Invitrogen
Spleen Phenotyping	HLA-DR	Qdot 605	TU36	Invitrogen
Spleen Phenotyping	CD8	Qdot 565	3B5	Invitrogen
Spleen Phenotyping	CD4	Qdot 655	S3.5	Invitrogen
Spleen Phenotyping	CD3	V500	SP34-2	BD
Spleen Phenotyping	CD3	Pacific Blue	SP34-2	BD
Spleen Phenotyping	CD20	Pacific Blue	2H7	BioLegend
Spleen Phenotyping	CD20	eFluor 450	2H7	eBioscience
Spleen Phenotyping	CD16	AF700	3G8	BD
Spleen Phenotyping	CD14	Pacific Blue	M5E2	BioLegend
Spleen Phenotyping	CD14	Qdot 655	TuK4	Invitrogen
Spleen Phenotyping	CD123	PE-Cy7	7G3	BD
Spleen Phenotyping	CD11c	APC	S-HCL-3	BD

Supplementary Table 2. Spleen Nanostring Codeset Sequences.

Gene Name	Target Sequence 5' - 3'
CCS	GGGCTGCATGGACTCCATGTCCATCAGTACGGGGACCTCACAAACAACTGCAACAGCTGTG<br>GGGACCACTTTAACCCTGATGGAGCATCTCATGGGGGCC
CD19	CCCAGACAGAGATATGTGGGTAGTGGACACGGGTCTGTTGTTGACCCGGGCCACAGCTCAA<br>GACGCTGGGAAGTATTATTGTCACCGTGGCAACTGGACC
GEM	ACTGCGAGGTGCTGGGAGAAGATACATATGAACGAACCCTGATGGTTGATGGGGAAAGTG<br>CAACGATTATACTCCTGGATATGTGGGAAAATAAGGGGGA
HAAO	ACAAGCTCATGCACCAGGAGCAGCTCAAAGTCATGTTCGTTGGAGGCCCCAATACCAGGAA<br>GGACTATCACATCGAAGAGGGTGAAGAGGTATTTTACCA
IDO1	GATGTTCATTGCTAAACATCTGCCTGATCTCATAGAGTCTGGCCAACTTCGAGAAAGAGTTG<br>AGAAGTTAGACATGCTCAGCATTGATCATCTCACAGAC
KMO	TGAAGAAACCTCGCTTTGATTACAGTCAGCAGTACATTCCTCATGGGTACATGGAGTTGACT<br>ATTCCACCTAAGAACGGAGATTATGCCATGGAACCTAA
KYNU	ACTCTATTTACATGACTGGGGAGTTGATTTTGCCTGCTGGTGTTCCTACAAGTATCTAAATGC<br>AGGCGCAGGAGGAATTGCCGGTGCCTTTATTCATGAA
QPRT	ATCTCCATGGGGATGCTGACCCAGGCGGCCCCAGCCCTTGATTTCTCCCTCAAGCTGTTTGCC<br>AAAGAGGCGGCTCCAGTGCCCAAAATCCACTGGTCCT
SIV17E-Fr	ATAATACTGTCTGCGTCATCTGGTGCATTCACGCAGAAGAGAAAGTGAAACACACTGAGGA<br>AGCAAAACAGATAGTGCAGAGACACCTAGTGGTGGAAAC
STK25	TGGAGGAGGCCGAGGATGAGATTGAGGACATCCAGCAGGAGATCACCGTCCTCAGCCAGT<br>GCGACAGCCCCTACATCACCCGCTACTTTGGCTCCTACCT

Supplementary Table 3. Colon and ileum Nanostring Codeset sequences.

Gene Name	Target Sequence 5' - 3'
HAAO	CAGCTCTGAGCAGTACAGAACAGGAAAGCCCATCCCTGACCAGCTTCTCAAGGAGCCACCAT TCCCTCTGAGCACACGATCCATCATGGAGCCCATGTCC
HPRT1	GAAGAGCTATTGTAATGACCAGTCAACAGGGGACATAAAAGTAATCGGAGGAGATGATCTC TCAACTTTAACTGGAAAGAATGTCTTGATTGTGGAAGAT
IDO1	GATGTTCATTGCCAAACATCTGCCTGATCTCATAGAGTCTGGCCAACTTCGAGAAAGAGTTG AGAAGTTAGACATGCTCAGCATTGATCATCTCACAGAC
KMO	AGAAATACTTTCCGGATGCCATCCCTCTAATTGGAGAGAAACTCCTGGTGCAAGATTTCTTCC TGTTGCCTGCCCAGCCCATGATATCTGTAAAGTGCTC
KYNU	TCTAGAAGCCAAAGCCTTCCCTTCTGATCATTATGCTATTGAGTCACAACTACAACTTCACGG ACTTAACATTGAAGAAAGTATGCGGATGATAAAGCCA
QPRT	CCAGGCGGCCCCAGCCCTTGATTTCTCCCTCAAGCTGTTTGCCAAAGAGGCGGCTCCAGTGC CCAAAATCCACTGGTCCTAAACCGGAAGAGGATGACAC
RPL13A	CGTACGCTGCGAAGGCATCAACATTTCTGGCAATTTCTACAGAAACAAGTTGAAGTACCTGG CTTTCCTCCGCAAGCGGATGAACACCAACCCTTCCAGA
RPS9	CGGCTGGTCCGCATTGGGGTGCTGGATGAGGGCAAGATGAAGCTGGATTACATCCTCGGCC TGAAGATCGAGGATTTCTTAGAGAGACGCCTGCAGACCC
SDHA	TGTCATAACTGCCTTTATACGCTTCTGCACTCCGGGGAAAAAGGAGTACATTGAAGGGAGAT TGGCACCCAGTGGCTGGTAGCTTGCCAGGAACCCAGTG

Supplementary Table 4. Median baseline levels of metabolites.

Tissue	TRP (μM)	KYN (μM)	3HK (μM)	QUIN (μM)	KYN/TRP Ratio x 100
Spleen	104.2	6.45	0.30	59.7	5.02
Brain	21.8	0.72	0.06	0.17	3.44
Plasma	18.2	2.26	0.08	2.47	11.0
CSF	2.73	0.28	0.03	0.09	5.53

Supplementary Table 5. Spleen samples used for metabolite and enzyme expression data.

Day p.i.	No. of Animals
Uninfected	5
4	6
7	6
10	5
14	6
21	6
35	6
42	9
Terminal	9
Total	58
